# Characterization of Motorcyclist Aggressive Driving Behavior in Urban and Suburban Environments: A Case Study of a Single Motorcyclist

**DOI:** 10.3390/s26113455

**Published:** 2026-05-30

**Authors:** Libânia Mendes, Andreia Teixeira, Rute Carvalho, Isabel Barroso, Jaime Sampaio, Vítor Rodrigues

**Affiliations:** 1 Department of Sports Science, Exercise and Health, University of Trás-os-Montes and Alto Douro, 5000-801 Vila Real, Portugal; libaniam@utad.pt (L.M.); rutecarvalho@utad.pt (R.C.); ajaime@utad.pt (J.S.); 2 Heart Research Community, Research Center in Sports Sciences, Health Sciences and Human Development (CIDESD), 5000-801 Vila Real, Portugal; vmcpr@utad.pt; 3 Elite Research Community, Research Center in Sports Sciences, Health Sciences and Human Development (CIDESD), 5000-801 Vila Real, Portugal; 4 School of Health, RISE-Health, Clinical Academic Center of Trás-os-Montes and Alto Douro (CACTMAD), University of Trás-os-Montes and Alto Douro, 5000-801 Vila Real, Portugal; imbarroso@utad.pt

**Keywords:** accelerometry, aggressive behavior, real-time analysis, road safety

## Abstract

**Highlights:**

**What are the main findings?**

- Environment-specific acceleration and braking thresholds differed substantially between the urban and suburban motorcycle routes.
- Most riding behavior was classified as normal, while suburban riding showed fewer but higher-intensity acceleration and braking events.

**What are the implications of the main findings?**

- Fixed acceleration thresholds derived from passenger cars may not accurately characterize motorcycle riding behavior across different riding environments.
- The standard deviation-based framework shows preliminary feasibility for motorcycle behavior classification but requires validation in larger and more diverse rider samples.

**Abstract:**

Aggressive riding behavior is a key contributing factor to road accidents, particularly in motorcycling, where rider dynamics directly influence vehicle stability and control. Despite growing interest in objective behavioral assessment, validated classification frameworks specific to motorcycles remain scarce in the literature. This pilot study investigated the feasibility of a standard deviation-based method for classifying aggressive riding behavior in a single experienced motorcyclist navigating two distinct environments: an urban route (UR) and a suburban national route (SNR). The participant completed two 20 min rides under real-world conditions. The UR was characterized by frequent accelerations, braking, speed bumps, and traffic lights, whereas the SNR features low traffic density and minimal interruptions. Longitudinal acceleration data were continuously recorded using a Vicon Blue Trident measurement unit mounted on the motorcycle seat. Drawing on the threshold principles established in automotive research, an environment-specific classification framework was developed to categorize riding events into normal, aggressive, and dangerous levels for both acceleration and deceleration maneuvers. The derived thresholds revealed pronounced environmental differences: UR thresholds (acceleration: 2.122 m/s^2^; deceleration: −2.134 m/s^2^) were approximately three times lower than those observed in the SNR (acceleration: 6.16 m/s^2^; deceleration: −7.09 m/s^2^). From more than four million recorded data points, approximately 88% of the riding behavior was classified as normal in both routes. In the UR, 9.27% of events were identified as aggressive and 4.37% as dangerous, compared with 7.27% aggressive and 5.35% dangerous events in the SNR. These preliminary findings suggest that environment-specific thresholds may be essential for accurately characterizing motorcycle riding behavior, and caution against the direct application of fixed automotive criteria to motorcycle safety analyses. All findings are specific to one rider on two routes and must not be extrapolated to other motorcyclists, vehicle types, or road contexts without replication.

## 1. Introduction

Road safety remains a major global public health issue. In 2021, road traffic accidents caused around 1.19 million deaths worldwide, with motorcyclists representing nearly 30% of all traffic-related fatalities [1]. Riding behavior is considered a primary factor in motorcycle crashes [2], highlighting the need for objective ways to assess behavior and support preventive strategies [3].

Riding behavior refers to how a person acts, responds, and makes decisions while operating a motorcycle. The literature usually classifies riding behavior into three behaviors categories: normal (safe), aggressive, and dangerous [4,5]. Normal riding involves consistent adherence to safe riding practices [6]. Aggressive riding is usually marked by excessive or inconsistent acceleration and braking, frequent speeding, and risky maneuvers [7]. Dangerous riding, involves deliberate violations of road safety and norms [8]. However, the distinction between aggressive and dangerous riding remains a methodological challenge, particularly for motorcycles, whose dynamics differ from those of cars [9].

In cars research, acceleration thresholds are already well established. Aggressive acceleration is defined between 3.5 and 7 m/s^2^ and dangerous acceleration ranges from 7 to 12 m/s^2^ [10,11]. Notwithstanding, motorcycles differ from cars in stability, load distribution, braking capacity, and rider posture [12]. Given these differences, directly applying passenger-vehicle thresholds to motorcycles is not methodologically appropriate. Research in motorcycle ergonomics also shows that posture, body dimensions, and musculoskeletal demands strongly influence rider performance and comfort [13]. Additionally, accelerometry methods provide a more objective and complete assessment of riding behavior than subjective tools alone [14].

Driving and riding behavior is also influenced by the road type and surrounding environment [15]. Urban roads usually involve dense traffic, with frequent intersections, lower speed limits, and speed bumps. In contrast, suburban roads often allow higher travel speeds, less congestion, and fewer interruptions [16]. These differences can affect rider decision-making and the likelihood of aggressive or dangerous behavior [17]. Regardless, most existing behavioral classification systems do not compare between urban and suburban contexts, limiting their transfer to the real world.

Traditional methods used in this research have relied mainly on self-reported questionnaires, demographic information, and observational approaches [18,19,20]. Although these methods provide valuable insights into rider attitudes and perceptions, they are limited by social desirability bias, low temporal resolution, and an inability to capture behavior in real time [9]. Questionnaire-based tools also face challenges related to internal consistency and external validity, especially across different riding settings [14].

Recent advances in inertial sensing technology have enabled objective, high-resolution data collection during real-world riding. Tri-axial accelerometers, such as the Vicon Blue Trident, continuously record acceleration across three axes (x, y, z), capturing the full dynamic motion of a motorcycle [21]. Inertial measurement unit (IMU) methods are increasingly being used in motorcycle research. Navratil et al. [22] showed that IMU data could classify rider behavior into four categories with about 80% accuracy using machine learning. Another study also reported that IMUs and accelerometers are one of the main tools used in naturalistic motorcycle studies, while noting the lack of validated motorcycle threshold systems [23]. Compared with GPS speed data, which may suffer from delays and positional inaccuracies, accelerometry provides a more direct and precise measure of instantaneous acceleration and braking behavior.

Despite these advances, several important gaps remain. First, most threshold-based behavioral classification models were developed exclusively for cars [10,11], making their direct use for motorcycles questionable given the clear differences in vehicle dynamics [12]. Second, although IMU systems have been used to detect critical riding events [21], no standardized, data-driven framework currently exists for defining motorcycle-specific thresholds across different environments. Third, relatively few studies have compared urban and suburban roads when analyzing aggressive riding, despite evidence that road type strongly shapes rider behavior and risky tendencies [15,17]. Passenger vehicle safety systems may not work correctly for motorcycles because of their dynamic characteristics [24] reinforcing the need for motorcycle-specific classification tools. This is especially important considering that motorcycles represent nearly 30% of global road fatalities [1], while many behavior models remain based on passenger vehicle assumptions.

This pilot case study addresses these gaps by applying a standard deviation (SD)-based, data-driven method to develop motorcycle-specific, environment-dependent behavioral thresholds using high-frequency IMU data collected in real-world conditions. Rather than assuming that car-based benchmarks can be transferred to motorcycles, this study utilized the observed distribution of riding behavior itself as the reference standard, an approach previously proposed in driving behavior research [9,22], but not yet systematically applied to motorcycle longitudinal acceleration data across different road environments. Data was collected from one experienced motorcyclist riding an urban route (UR) and a suburban national route (SNR) using the Vicon Blue Trident IMU. These findings will provide preliminary evidence that this method is feasible and offers a replicable framework for future studies with a large sample.

This pilot study tested two primary hypotheses: (1) acceleration and deceleration thresholds derived using the SD method would be lower in the UR than in the SNR, and (2) the proportion of aggressive or dangerous riding events would be higher in the urban environment because of its greater complexity, density, and frequency of disruptions.

## 2. Materials and Methods

### 2.1. Participant

The case study was conducted on a single experienced female motorcyclist (age 36 years; riding experience: 20 years of daily commuting use). The participant had a valid motorcycle license and reported no self-reported musculoskeletal, neurological or cardiovascular conditions that may affect riding performance or reaction time. The participant used the study motorcycle regularly to become familiar with its braking response and handling characteristics. The main objective of this pilot case study was to validate the threshold derivation approach, and for this purpose, the participant design was adopted. The present study was conducted in accordance with the ethical principles of the Declaration of Helsinki. Prior to data collection, written informed consent was obtained from the participant following a full explanation of the study’s purpose, procedures, and data storage. The protocol received institutional ethical (Doc96-CE-UTAD-2024).

### 2.2. Measured Motorcycle

**Figure 1:**
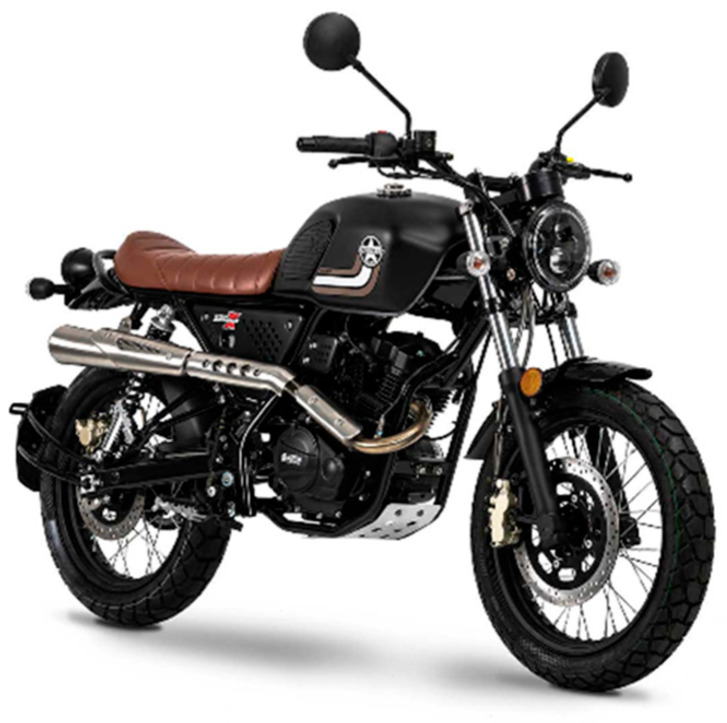
UM RENEGADE SCRAMBLER X NAKED motorcycle.

The motorcycle used in the present study was a UM RENEGADE SCRAMBLER X NAKED (Figure 1).

### 2.3. Experimental Procedure

Two routes were selected to represent the two different riding environments that experienced urban motorcycle riders navigate in everyday practice. Routes were selected according to the following criteria: (1) the representative nature of each route to its environmental category, based on infrastructure, speed regime, and traffic density; (2) the total riding duration per route was matched to approximately 20 min to standardize exposure time and reduce fatigue confounding of acceleration behavior [24]; and (3) both routes were familiar to the participant, to reduce the effect of navigational uncertainty on riding dynamics, in accordance with the naturalistic riding methodology that prioritizes observation of habitual, ecologically valid behavior [22].

The UR was in the municipality of Vila Real, Portugal, and was characterized by a speed limit of 50 km/h, a high frequency of signalized intersections, pedestrian crossings, roundabouts, road surface irregularities, and variable traffic density during data collection. These characteristics are in line with current definitions of urban road environments in the motorcycle safety literature [15,16], leading to periodic stop-and-go dynamics for the rider.

The SNR corresponded to a classified national road in Vila Real (Portugal) with a speed limit of 90 km/h, few intersections, low traffic density, and long patches without interruptions. Such roads allow higher operating speeds and more freedom of longitudinal maneuver, resulting in acceleration and deceleration profiles qualitatively different from those of the urban environment [15,17]. Road type has been a key predictor of motorcyclist aggressive maneuvering in naturalistic riding research, with different behavioral distributions in urban and suburban environments.

The participant performed the two riding sessions on separate occasions. Sessions were planned outside the peak hours to avoid rush hour traffic congestion affecting the riding dynamics. For both sessions, weather conditions were characterized by clear skies and low humidity, with ambient temperatures remaining in seasonal range typical of the region. The participant was instructed to ride in their usual way, without instructions concerning speed, acceleration, or braking style, in accordance with the naturalistic riding approach.

### 2.4. Acceleration Data

**Figure 2:**
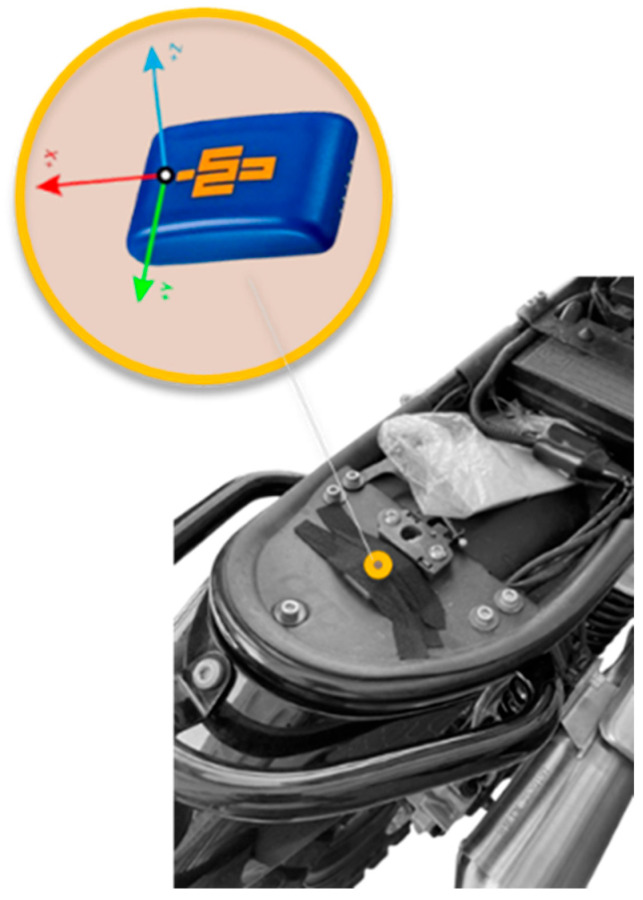
Vicon Blue Trident positioned on the motorcycle seat.

Data were collected using Vicon Blue Trident tri-axial accelerometers (Vicon Motion Systems Ltd., Oxford, UK) mounted on the motorcycle seat. Each IMUs contains: a low-g and high-g tri-axial accelerometer (±16 g at 1125 Hz; ±200 g at 1600 Hz, respectively), gyroscope (±2000 deg/s at 1125 Hz) and magnetometer (±4900 µT at 100 Hz). The longitudinal acceleration component (ax) was extracted and pre-filtered using standard signal processing techniques. The IMU was secured with Kinesio tape and manufacturer provided straps, ensuring no discomfort for the participant. Data were synchronized and recorded using Capture.U software (Version 1.4.1, Vicon Motion Systems Ltd., Oxford, UK) on an Apple iPad, 64 GB (Apple Inc., Cupertino, CA, USA (Figure 2)).

### 2.5. Data Pre-Processing

Data collection was followed by the pre-processing of raw acceleration data to prepare them for the subsequent analysis. The two-stage framework of Abdulwahid et al. [25] was followed for this. The raw data was preprocessed after the completion of the data collection to make it ready for use [25]. The preprocessing pipeline for the longitudinal acceleration component (*ax*) consisted of three consecutive steps: moving average smoothing, mean centering and orientation correction. This pipeline is detailed in the following.

Stage 1: The raw acceleration signal was filtered with a symmetric moving-average filter of window size *k* = 5 samples. The filtered value ã*i* for each sample *i* was calculated as the mean of the two following samples:

ãi =(1/5)×Σai+j,j=−2,−1,0,+1,+2

The moving average filter was used to suppress high-frequency noise measurement due to micro-vibrations of the road surface and chassis resonance that cannot be attributed to purposeful riding behavior. At the sampling frequency of 200 Hz of the Vicon Blue Trident, a window of *k* = 5 results in an effective cutoff frequency of approximately 12.7 Hz (fc = fs/(π × *k*)) which preserves the relevant frequency range of deliberate acceleration and braking maneuvers (typically below 5 Hz) and suppresses higher frequency noise [25]. The window size *k* = 5 was chosen as the minimal size providing a visually stable signal envelope while not distorting the onset of fast braking events.

Stage 2: After smoothing, sensor bias was removed by mean centering for recalibration [25]. For each filtered sample, the route-specific mean (μR) was subtracted:

a∗i=ãi−μR, where μR=(1/N)×Σãi

Mean centering serves two purposes in line with the rationale for preprocessing used by Abdulwahid et al. [25]. It removes the static DC offset of the MEMS accelerometer electronics and aligns the central tendency of the signal with zero, the behavioral reference point at which net acceleration and net deceleration are equal. This alignment is a necessary condition for the symmetric “$\pm n\sigma$” threshold formulation discussed in Section 2.7. Mean centering was applied globally to each full route to preserve the distributional properties necessary for the SD-based threshold derivation.

Stage 3: Orientation correction was applied according to the mounting configuration of the sensor [25], to ensure that positive acceleration values represented forward movement (acceleration) and negative values represented braking or deceleration events. The Vicon Blue Trident IMU was mounted on the motorcycle seat with the longitudinal axis aligned with the forward axis of the vehicle, although the practical constraints on the seat geometry may introduce a small mount angle offset θ, projecting a gravitational component of g × sin(θ) onto the longitudinal channel. The mean acceleration recorded during the stationary pre-session calibration window (a_static_) was subtracted from the preprocessed signal to correct for this offset:

a∗∗i=a∗i−astatic

This correction guaranteed the directional consistency of the signal on both routes, in line with the data cleaning and preprocessing procedure described by Abdulwahid et al. [25].

### 2.6. Data Analysis

Given the single-participant pilot design, the analysis was exploratory and descriptive in nature. No statistical tests comparing between-participant means were conducted, as such tests are inappropriate with n = 1. The analysis was performed with two goals: (1) to obtain environment-specific acceleration and deceleration thresholds using the SD-based procedure described in Section 2.7, and (2) to classify all recorded data points based on the obtained thresholds, reporting proportional counts of normal, aggressive and dangerous samples separately for acceleration and deceleration in each route. Descriptive statistics (mean, SD) were calculated for the longitudinal acceleration signal per route before the threshold derivation. To test the normality assumption behind the SD approach, the symmetry of the threshold values for acceleration and deceleration around zero was qualitatively checked. All data processing and analysis were performed in RStudio (v4.5.1; Posit Software, PBC, Boston, MA, USA) using the base R statistical environment.

### 2.7. Threshold Formulation for Behavior Classification

Following the methodological framework of Abdulwahid et al. [25], a data-driven, SD-based approach was adopted to derive motorcycle-specific behavioral thresholds for each route environment. Several researchers have investigated the acceleration and deceleration limits of vehicles in relation to accident causality and risk [10,11]; however, specific limits for motorcycles are not well established in the literature and cannot be directly derived from automotive benchmarks [25]. In the present study, the threshold derivation procedure was applied to the preprocessed longitudinal acceleration data of each route independently, yielding environment-specific classification boundaries.

The methodology assumes that the most frequently observed acceleration behavior is normal riding, while the less frequently observed behaviors are aggressive and dangerous [25]. This assumption is in accordance with the principles of the normal distribution: data within one SD (σ) from the mean (μ) constitute about 68.2% of observations, data within two SD account for about 95.4% and data within three SD account for about 99.7% [25,26]. In mathematical terms:

Pr(μ−1σ≤X≤μ+1σ)≈68.27%

Pr(μ−2σ≤X≤μ+2σ)≈95.45%

Pr(μ−3σ≤X≤μ+3σ)≈99.73%

where Pr() is the probability function, X is the observation of the normally distributed random variable, μ is the mean of the distribution and σ is the SD [25].

In the present study, the ±1σ and ±2σ thresholds are applied symmetrically around zero for both positive (forward acceleration) and negative (deceleration/braking) directions of the mean-centered signal. This adaptation is justified because the Vicon Blue Trident IMU provides a direct measurement of the signed longitudinal acceleration, incorporating both thrust and braking dynamics in a single continuous signal.

#### Threshold Calculation Procedure

For acceleration events, the thresholds are calculated as follows:

Step 1: Calculate the mean acceleration (μ) across all preprocessed data points in route R:

$$\mu =(1/n)\times \Sigma ni=1A_{i}$$

where A_i_ = acceleration value at sample i; n = total number of samples in the route.

Step 2: Calculate the SD (σ) of the acceleration signal:

$$\sigma =\surd [(1/(N-1))\times \Sigma Ni=1{\left(X_{i}-\bar{X}\right)}^{2}]$$

where N = total number of acceleration samples; X_i_ = value of acceleration at sample *i*; $\bar{X}$ = mean acceleration (m/s^2^).

Step 3: Define the three-tier threshold boundaries for acceleration, following SD rule [25]:

Normal Acceleration: a_i_ ≤ μ + 1σ

Aggressive Acceleration: μ + 1σ < a_i_ ≤ μ + 2σ

Dangerous Acceleration: a_i_ > μ + 2σ

where a = longitudinal acceleration value (m/s^2^). The most frequently observed behavior, values within ±1σ of the mean, is labelled as normal, the less frequent band between ±1σ and ±2σ as aggressive, and the least frequent tail beyond ±2σ as dangerous.

The same four-step procedure was applied to deceleration data (negative acceleration values):

Step 1: Calculate mean deceleration (μ) across all preprocessed negative acceleration samples in route R:

$$\mu =(1/n)\times \Sigma ni=1A_{i}$$

where A_i_ = deceleration value for driving sample *i* (m/s^2^); n = number of deceleration samples.

Step 2: Calculate the SD (σ) of the deceleration signal:

$$\sigma =\surd [(1/(N-1))\times \Sigma Ni=1{\left(X_{i}-\bar{X}\right)}^{2}]$$

where N = number of deceleration samples; X_i_ = value of deceleration at sample *i*; $\bar{X}$ = mean deceleration (m/s^2^).

Step 3: Define the three-tier threshold boundaries for deceleration, applying the symmetric negative counterpart rule [25]:

Normal Deceleration: d_i_ ≥ μ − 1σ

Aggressive Deceleration: μ − 2σ ≤ d_i_ < μ − 1σ

Dangerous Deceleration: d_i_ < μ − 2σ

where d = longitudinal deceleration value (m/s^2^; negative by convention). The directionality of the inequalities is reversed relative to acceleration because more extreme deceleration corresponds to more negative values: a deceleration value below μ − 2σ is more dangerous than one above it.

### 2.8. Operational Definition of Riding Events

In this study, a riding event is defined as an individual data point from the preprocessed acceleration time series in which the signal value exceeds a route-specific behavioral threshold, following the approach of another study [25]. Abdulwahid et al. [25] operationalized events as per-second GPS speed samples, counting each sample independently and classifying it as normal, aggressive, or dangerous depending on whether it fell within the normal band (≤μ + 1σ), the aggressive band (μ + 1σ to μ + 2σ), or the dangerous band (>μ + 2σ). For the present study, the same sample-level definition of an event was applied, though adapted to the higher temporal resolution of the IMU, with each data point recorded at 200 Hz classified independently using the route-specific threshold boundaries derived in Section Threshold Calculation Procedure.

The driving profile is constructed from the continuous accumulation of classified samples over the full trip, rather than from the discrete segmentation of maneuvers. The total number of acceleration events and deceleration events reported in Section 3 is equivalent to the sum of individual data points labelled aggressive or dangerous, respectively. Event counts and proportional distributions are reported separately by each route and by acceleration and deceleration direction.

## 3. Results

### 3.1. Cut-Off Values

The route-specific threshold values were derived using the SD-based procedure described in Section 2.7 and applied to the preprocessed longitudinal acceleration signal for each route separately. As shown in Table 1, the classification thresholds for acceleration and deceleration events are defined in both the UR and SNR. It should be noted that all event counts reported in this section are based on the number of individual IMU samples where the acceleration values exceeded the respective behavioral thresholds, consistent with the operational definition given in Section 2.8. The UR presented lower thresholds for acceleration and deceleration, and the SNR presented higher threshold values. This result is indicative of the larger magnitudes of acceleration and braking observed across the SNR.

### 3.2. Events Distribution

The analysis results indicated that most driving events, both in acceleration and deceleration, were normal for both routes. Furthermore, several aggressive and dangerous events were identified, emphasizing the significance of driving maneuvers that affect safety (Table 2).

The near balance of acceleration and deceleration events counts within each route provides an additional qualitative check on the internal consistency of the SD approach.

**Figure 3:**
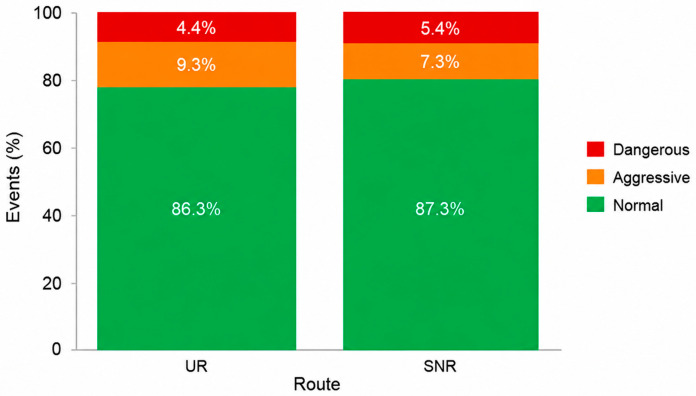
Percentage distribution of events in the two routes.

A total of 1854.716 events were detected on the UR. Normal accelerations were observed more often (86.3% of the acceleration events) compared to aggressive and dangerous accelerations (9.3% and 4.4%, respectively) (Figure 3). A similar tendency was observed in deceleration events, with 803.977 normal decelerations, 85.451 aggressive decelerations, and 38.696 dangerous decelerations. These values are consistent with the stop-and-go dynamics of the urban environment, where each acceleration phase is followed by a corresponding deceleration phase.

The SNR showed a larger total number of detected events (2252.540) than the UR, which can be explained by the longer uninterrupted segments and elevated operating speeds of the suburban context, producing more total data points per unit time. Normal acceleration patterns were predominant (1005.474 events); however, aggressive (95.642 events) and dangerous (64.103 events) acceleration events were observed more frequently than on the UR. By contrast, normal decelerations decreased (962.926 events), while dangerous decelerations (56.339 events) were higher than those found on the UR (38.696 events) (Table 2). The higher frequency of aggressive and dangerous accelerations on the SNR may be related to increased travel speeds, overtaking maneuvers, and longer uninterrupted patches of driving.

### 3.3. Hypothesis Assessment

The pilot case study established two main hypotheses. The first hypothesis, by comparing the SD method threshold values between UR and SNR, was confirmed. The UR showed an aggressive acceleration threshold of 2.122 m/s^2^ which was lower than in the SNR (6.161 m/s^2^), and the same pattern appeared with deceleration thresholds (UR: −2.134 m/s^2^; SNR: −7.088 m/s^2^). The dangerous thresholds showed the same pattern across both environments. These results are consistent with the notion that urban riding dynamics are mechanistically constrained relative to suburban riding, resulting in lower SD-derived thresholds.

The second hypothesis evaluated whether a greater proportion of riding events classified would be deemed aggressive or dangerous in the UR compared to the SNR. The UR led to a greater share of aggressive acceleration events (9.3%) than the SNR (8.5%), as well as a higher proportion of aggressive deceleration events (UR: 9.2%; SNR: 7.1%), consistent with the expectation that urban environments involve more frequent threshold exceedances. However, a different pattern emerged for dangerous events: the SNR showed a higher percentage of dangerous acceleration events (5.7%), compared to the UR (4.6%), and for deceleration, the SNR again exceeded the UR (5.8%; UR: 4.2%). This partial disconfirmation can be mechanistically interpreted as follows: urban riding generates more frequent low- and moderate-thresholds exceedances, whereas suburban riding results in fewer exceedances, but with higher intensity, making them more likely to reach the dangerous category. These findings suggest that the two environments present qualitatively different risk profiles.

## 4. Discussion

This pilot case study aimed to classify motorcycle riding behavior as normal, aggressive, or dangerous using the SD method and to determine its prevalence in two real-world environments: urban and suburban. In this single-participant pilot study, the results suggest that riding thresholds may need to be adapted to the road environment; this finding requires replication in larger samples before it can be considered generalizable. In the UR, thresholds were lower than in the SNR, which is consistent with both study hypotheses. This means that the same riding action may be classified as aggressive in one environment and normal in another.

For example, the aggressive acceleration threshold was 2.122 m/s^2^ in the UR and 6.161 m/s^2^ in the SNR. Aggressive braking showed a similar pattern (−2.134 vs. −7.088 m/s^2^). This reflects the very different physical demands the rider is exposed to in the two environments. In the UR, the ride is continually interrupted by the 50 km/h speed limit, traffic lights, pedestrian crossings, roundabouts, and speed bumps. Dense urban infrastructure has been shown to directly limit attainable speeds in motorcycles [15,17].

Two main mechanisms can explain the lowered thresholds in the UR. Stopping points are often close together, and braking in urban settings is generally progressive rather than abrupt, as riders continuously adjust to stop-and-go traffic patterns [16]. These factors combined compress the overall distribution of acceleration values and naturally lead to lower SD-based thresholds. Conversely, the SNR had a speed limit of 90 km/h, less traffic density, and longer uninterrupted sections of road. This meant the rider could sustain higher speeds for longer periods, causing greater changes in speed when accelerating and requiring stronger braking when slowing down. Naturalistic riding studies confirm that higher magnitude acceleration events occur more frequently on open roads with longer sustained speeds [21,23]. This provides a pragmatic explanation as to why thresholds cannot simply be swapped between the two routes, and why fixed thresholds that ignore the road environment would likely misclassify rider behavior in at least one setting. In practical terms, the aggressive events detected in the UR are most likely linked to acceleration bursts from rest at traffic lights, sudden braking before pedestrian crossings or speed bumps, and dynamic lane adjustment at roundabouts, maneuvers that are frequent but carried out within a low-speed envelope. The dangerous events in the UR likely correspond to the few instances when the rider had to brake harder than expected, such as when a vehicle or pedestrian suddenly entered their path.

The thresholds found in the UR (aggressive: 2.122 m/s^2^; dangerous: >4.244 m/s^2^) fall within the normal acceleration range typically reported for passenger cars (1.5–3.5 m/s^2^) [10,11]. This has an important implication: applying thresholds derived from car data to motorcycle data in urban areas would classify riding events that are relatively aggressive for the context as normal, rendering the system insensitive in environments with the highest crash risk for motorcyclists [17]. In contrast, the thresholds for the SNR (aggressive: 6.161 m/s^2^) fell within the aggressive range commonly reported for passenger cars (3.5–7.0 m/s^2^), and the threshold for dangerous deceleration (−10.817 m/s^2^) exceeded the dangerous braking threshold often used in car studies (−9.0 m/s^2^) [10,11]. This may reflect the superior power-to-weight ratio and braking ability of motorcycles relative to passenger cars [12]. On the SNR, the lower frequency and higher magnitude of threshold exceedances are consistent with events expected in a national road: string accelerations from higher speeds or braking harder when approaching an intersection or slower vehicle. Events are fewer in number but involve more kinetic energy and therefore involve a greater risk for injury in the event of an error. Together, these comparisons indicate that thresholds developed for cars are based on a different type of operating under different mechanical and environmental conditions; their direct application to motorcycles is methodologically incorrect.

The conceptual link between the data and behavioral risk requires justification. Under SD classification, this link rests on the assumption that a rider’s habitual acceleration and deceleration patterns represent their characteristic safe operation. Events within ±1σ of the mean correspond to maneuvers that the rider routinely and comfortably performs; events between ±1σ and ±2σ are markedly less frequent and may reflect deliberate effort (such as harder braking or sharper acceleration) rather than routine control inputs; and events beyond ±2σ are the most infrequent extremes, which in real riding tend to coincide with emergency responses, loss of anticipation, or high-risk maneuvers. This rationale aligns with a previous framework [25], which demonstrated how the SD-derived “aggressive” category captures acceleration events falling outside habitual controlled riding behavior. Nonetheless, a rider who habitually brakes hard at every stop would generate higher thresholds and therefore have fewer events classified as aggressive, even if their overall riding style is more forceful. This limitation is acknowledged and reinforces the need for future studies incorporating independent behavioral or safety-outcome measures.

Approximately 88% of all recorded data for both routes were classified as normal riding. This is consistent with the assumptions of the SD method and with previous naturalistic riding studies showing that riding is predominantly safe [9,25]. The remaining 12% of events showed different patterns across environments. The UR had a higher percentage of aggressive events (9.27% vs. 7.27%) but a lower percentage of dangerous events (4.37% vs. 5.35%) compared to the SNR. This is mechanically plausible because urban traffic repeatedly generates moderate accelerations and braking due to stop-and-go movement; such events frequently exceed the aggressive thresholds but are seldom dangerous because speeds remain relatively low. In the SNR, long periods of steady-speed riding were predominantly classified as normal. However, when exceedances did occur—such as during strong braking from a higher speed—they were larger in magnitude and therefore more likely to be classified as dangerous [3,27]. The findings suggest that safety risks may differ between environments: the UR involves more frequent but less severe events, while the SNR may involve less frequent but severe ones.

This pilot case study also highlighted several strengths of the SD method. First, the distribution of results was statistically consistent, with most riding classified as normal and fewer samples falling into the more extreme categories. Secondly, the thresholds were physically meaningful and consistent with the known characteristics of each route. Third, the acceleration and braking thresholds were consistent in direction, supporting the internal logic of the method. These features distinguish the SD approach from fixed-threshold systems, which use the same threshold regardless of road context [9,28], and from machine learning approaches that achieve high classification accuracy under controlled conditions [22] but require labeled training data and greater computational resources. For feasibility-oriented pilot studies, the SD approach offers a more transparent alternative.

It should be noted that the SD method identifies events that are statistically unusual relative to the rider’s own acceleration distribution; it does not directly confirm that these events represent aggressive or dangerous riding in a behavioral sense. A value beyond ±2σ indicates extreme acceleration relative to this rider’s norm; whether such extremes correspond to genuinely risky maneuvers requires corroboration through observational data, expert labeling, or linkage to safety outcomes in future work.

Overall, the results of this pilot case study indicate that the SD-based data-driven threshold method is a feasible and transparent method to classify motorcyclist riding behavior by road environment, providing a preliminary alternative to the direct application of car-based thresholds. In this case, the method produced physically meaningful, internally consistent, and route-consistent thresholds. However, these are proof-of-concept results, not formal validation. The extent to which these thresholds and environmental patterns generalize to other riders, motorcycle types, or road contexts remains unknown and requires investigation in larger, more diverse naturalistic studies.

### Limitations and Future Directions

The present study has several limitations. First, the single-participant design limits the generalizability of the observed thresholds and behavioral patterns. All results reflect this specific rider’s experience and riding style and must not be extrapolated generally. Second, the lack of controlled traffic flow data means that the observed differences may have been caused by variations in traffic conditions rather than solely by differences in road type. Third, thresholds were derived from the data of a single rider; future studies should compare these thresholds with those obtained from expert evaluations or machine learning classifications trained on larger datasets. A further limitation is that the study did not examine whether aggressive or dangerous events corresponded to real-world safety outcomes, such as near-collisions.

To address these limitations, future research should aim to replicate this study with larger and more heterogeneous samples, including diversity in age, gender, riding experience, and motorcycle type. Further work should also examine the influence of environmental factors such as traffic congestion, illumination, and weather conditions on longitudinal acceleration behavior. Comparing motorcyclist behavior under different loading conditions, such as a solo rider, rider with a passenger, or rider with cargo, would also be a valuable direction.

## 5. Practical Applications

The preliminary results of this pilot case study suggest that the environment-specific threshold frameworks may be practically useful for improving motorcycle safety, but these findings must be verified with a larger and more heterogeneous sample of riders before any applied use can be considered. When applied to motorcycle acceleration data, a fixed threshold system has a significant limitation: it is either too insensitive for urban environments or poorly calibrated for suburban riding conditions. The SD-based approach could support the development of motorcycle monitoring systems and rider assistance technologies that automatically adjust behavioral thresholds according to the detected road environment. This could minimize false alerts in city traffic while still detecting truly hazardous situations on faster, more open routes. Such context-aware systems could offer riders and fleet operators more valuable and actionable insights than conventional static threshold systems, provided the approach is first validated in a broader and more diverse rider population, which the present pilot study does not establish. In addition, the methodological framework could be applied to other dimensions of riding behavior, such as lateral acceleration cornering, speed relative to posted limits, and following distance maintained behind other vehicles, again subject to validation on larger rider samples.

## 6. Conclusions

This pilot case study examined the riding behavior of a single experienced motorcyclist in two real-world environments: a UR and an SNR. A data-driven approach based on SD was used to classify riding behavior as normal, aggressive, or dangerous. The UR showed lower thresholds for aggressive acceleration and deceleration (2.122 m/s^2^ and −2.134 m/s^2^, respectively) than the SNR (6.161 m/s^2^ and −7.088 m/s^2^, respectively). This pattern is consistent with the hypothesis that the road environment influences riding behavior. Most riding was classified as normal, with fewer aggressive events (UR = 9.27%, SNR = 7.27%) and dangerous events (UR = 4.37%, SNR = 5.35%). These results suggest that the SNR may be less frequently disruptive overall, but when threshold exceedances do occur, they are associated with stronger acceleration and braking.

These preliminary findings provide initial proof-of-concept support for the SD-based threshold method as a plausible approach to classifying riding behavior by road context. The method demonstrated internal consistency and aligned with patterns reported in the previous literature, although the study was limited to one rider and two routes, and must not be considered a formal validation. It establishes a replicable methodology basis for future larger-scale studies. If research with larger rider samples confirms its generalizability, subsequent work could explore the use of this framework in rider safety assistance systems, real-time behavioral feedback tools, and motorcycle safety interventions.

Road safety research increasingly recognizes that context-aware, data-driven threshold systems are more realistic than fixed thresholds, since riding and driving behavior naturally vary with road type, speed, and traffic density. It must be acknowledged, however, that statistical extremity serves as a proxy for behavioral risk rather than a direct measure of it. Future research should verify whether SD-derived classifications of “aggressive” and “dangerous” behavior correspond to events that observers or riders themselves would identify as risky. The SD-based approach shows preliminary methodological promise for classifying motorcycle riding behavior within this single-participant pilot context but must be treated as a preliminary proposal until tested on larger and more diverse rider samples.

## Figures and Tables

**Table 1:** Classification thresholds by driving environment.

	Acceleration (m/s^2^)	Deceleration (m/s^2^)
Urban Route	Normal: a_i_ ≤ 2.122	Normal: a_i_ ≥ −2.134
	Aggressive: 2.122 < a_i_ ≤ 3.126	Aggressive: −3.151 ≤ a_i_ < −2.134
	Dangerous: a_i_ > 3.126	Dangerous: a_i_ < −3.151
Suburban National Route	Normal: a_i_ ≤ 6.161	Normal: a_i_ ≥ −7.088
	Aggressive: 6.161 < a_i_ ≤ 9.187	Aggressive: −10.817 ≤ a_i_ < −7.088
	Dangerous: a_i_ > 9.187	Dangerous: a_i_ < −10.817

**Table 2:** Driver profiling according to route type.

Route		UR (n)	SNR (n)
Accelerations	Normal	797.855	1005.474
	Aggressive	86.473	95.642
	Dangerous	42.264	64.103
Decelerations	Normal	803.977	962.926
	Aggressive	85.451	68.056
	Dangerous	38.696	56.339
Total		1854.716	2252.540

UR, Urban Route; SNR, Suburban Nation Route.

## Data Availability

The raw data supporting the conclusions of this article will be made available by the authors on request.

## Abbreviations

The following abbreviations are used in this manuscript:

IMU	Inertial Measurement Unit
SD	Standard deviation
SNR	Suburban national route
UR	Urban route
